# Psychological Resilience and Quality of Life in Mycosis Fungoides Patients: A BIO-MUSE Study

**DOI:** 10.2340/actadv.v106.adv-2025-0211

**Published:** 2026-06-29

**Authors:** Angelica Johansson, Katarina Velickovic, Emma Belfrage, Kristina Drott, Per Johnsson, Sara Ek

**Affiliations:** 1 Department of Immunotechnology, Faculty of Engineering, Lund University, Lund, Sweden; 2 Department of Health Sciences, Faculty of Medicine, Lund University, Lund, Sweden; 3 Division of Dermatology and Venereology, Department of Clinical Sciences, Lund University, Skåne University Hospital, Lund, Sweden; 4 Division of Medical Oncology, Department of Clinical Sciences, Faculty of Medicine, Lund University, Lund, Sweden; 5 Department of Psychology, Lund University, Lund, Sweden; 6 CREATE Health Translational Cancer Center, Lund University, Lund, Sweden

**Keywords:** cutaneous T cell Lymphoma, dermatology, mycosis fungoides, psychological resilience, quality of life

## Abstract

Cutaneous T-cell lymphoma is a chronic disease with a highly heterogeneous and unpre-dictable clinical course, and the impact on psychological resilience is still unknown. The primary aim was to investigate psychological resilience in cutaneous T-cell lymphoma patients over time and to relate it to dermatology-related quality of life (QoL) metrics. Psychological investigations were evaluated in 45 patients with mycosis fungoides included in the BIO-MUSE study. Psychological resilience was assessed using the Connor–Davidson Resilience Scale (CD-RISC), and dermatology-associated QoL was assessed with the Dermatology Life Quality Index (DLQI) and numeric rating scales for itch and sleep disorders. The resilience of the mycosis fungoides patients was further compared to a Swedish general population cohort comprising more than 2,500 individuals. This study demonstrates that mycosis fungoides patients in the BIO-MUSE cohort, where 39 of 45 cases had early-stage disease, exhibit a high and stable resilience over 1 year of the disease. The CD-RISC score is higher than in a Swedish general population cohort and in a subgroup of that cohort with psoriasis or eczema. Furthermore, the DLQI was consistently low, indicating a higher QoL in the BIO-MUSE cohort than in published data for similar patient populations.

**SIGNIFICANCE**

Cutaneous T-cell lymphoma is a rare cancer arising from T-cells in the skin. Mycosis fungoides is the most common subtype of cutaneous T-cell lymphoma and has an indolent course, with patients experiencing dermatological symptoms for a long time. This study represents an essential step toward understanding psychological resilience in patients with mycosis fungoides, a topic that has not been previously explored. The findings show that mycosis fungoides patients in a Swedish cohort exhibit high resilience and quality of life. These results can help inform patients and guide interventions to maintain high resilience and quality of life among individuals with mycosis fungoides.

Cutaneous T-cell lymphoma (CTCL) is a rare malignancy that arises from skin-infiltrating CD4+ T cells. Mycosis fungoides (MF) is the most common subtype of CTCL, with 6–9 cases per million people, accounting for approximately 60% of all cases (1). MF often has an indolent course, and many patients have a normal life expectancy (2). Staging of MF is based on the extent of involvement of skin, lymph nodes, blood and visceral organs. At an early stage (IA–IIA), the disease presents as patches or plaques, ranging from limited to generalized involvement with favourable prognosis, and a 5-year overall survival (OS) rate of 84%–94% (3, 4). However, about 25% of cases eventually progress to advanced-stage disease, presenting as widespread patches, plaques, tumours, erythroderma or systemic disease, with a 5-year OS of approximately 18%–47% (IVB) (5).

Patients with MF experience a wide range of dermatological symptoms, such as pruritus, pain and scaly, dry skin, over prolonged periods. With limited curative treatment options available, most therapies focus on preventing disease progression and restraining the symptoms. Treatment-related side effects and symptoms can significantly impact health-related quality of life (HRQoL) (6–8). In skin diseases such as MF, dermatology-specific HRQoL assessments are available, including the Dermatology Life Quality Index (DLQI) (9, 10).

A patient’s ability to cope with stressors, e.g. related to diagnosis, symptoms or treatment side-effects, and thereby maintain a high HRQoL, has been linked to psychological resilience (11, 12). Psychological resilience is the capacity to adapt and cope with adversity or unexpected life changes. In 2003, Connor and Davidson developed the Connor–Davidson Resilience Scale (CD-RISC), a widely used self-reported measure of resilience (13, 14). In 2020, CD-RISC was assessed in a Swedish general population cohort (15), and it was concluded that CD-RISC is a valid tool for assessing psychological resilience in clinical settings in Sweden.

Given that MF is a visible, indolent and long-lasting disease, it is essential to understand how psychological resilience is affected in these patients. Stressors may arise not only at diagnosis but also throughout the course of the disease. With no standard psychological counselling provided to these patients, the disease could potentially impact their psychological resilience.

In this study, psychological resilience and dermatological-associated QoL were evaluated in 45 MF patients enrolled in the prospective longitudinal BIO-MUSE trial (“Predictive and prognostic biomarkers in patients with mycosis fungoides and Sézary syndrome”) (16). Psychological investigations at baseline were compared with those obtained at 1-year follow-up. Furthermore, psychological resilience was related to a Swedish general population cohort (17).

This valuable dataset from the BIO-MUSE study provides the first evaluation of psychological resilience in MF patients. It has the potential to inform future clinical interventions to support psychological resilience and QoL in these patients.

## MATERIALS AND METHODS

### Patient cohorts

The prospective translational study, BIO-MUSE, was approved by the Swedish Ethical Review Authority (2019-15130), complemented with psychological investigations in 2021 (2021-06742-02). All patients signed an informed consent before inclusion in the BIO-MUSE trial.

Inclusion criteria to participate in the BIO-MUSE trial are age between 18 and 100 years, MF or SS with stage IA-IVB, and World Health Organization (WHO) performance status 0–3. Patient enrolment in the BIO-MUSE trial began in April 2021 and continues to this day. Patients in the BIO-MUSE trial are monitored for 3 years, with data collected on both biological and psychological measures (16). Data were collected at baseline and at 1-year follow-up for the patients included in the BIO-MUSE study for psychological investigations. Baseline of this study refers to the date when the first CD-RISC was completed and does not, in all cases, correspond to the date of enrolment in the BIO-MUSE trial. Patients included in the resilience study from the BIO-MUSE trial are referred to as *resilience cohort A*.

The patient cohort in the BIO-MUSE trial, i.e. resilience cohort A, was complemented with an additional MF patient cohort included anonymously via postal correspondence, referred to as *resilience cohort B*. The inclusion criteria were the same as for the BIO-MUSE trial, except that only patients with presumably early-stage MF were included. Information on the stage of disease at enrolment for these patients was not available, but since they had not been referred to the department of oncology, which is routine for advanced-stage patients at Skåne University Hospital, they were assumed to be early-stage (IA–IIA). Participants in resilience cohort B only participated in the psychological investigations, and no biological sampling was performed. Enrolment of the resilience cohort B took place in spring 2024, and the patients were asked to join the 1-year follow-up investigations during spring 2025.

The complete resilience cohort, comprising both *resilience cohort A* and *resilience cohort B*, is referred to as the *BIO-MUSE resilience cohort*.

To provide a reference population, data on CD-RISC, age, gender and disease status were also collected from a Swedish general population cohort from the BIG3 project (15, 17). The BIG3 project is an observational, prospective longitudinal cohort study in Sweden with an overall aim to understand the disease mechanisms underlying the 3 major public health diseases: chronic obstructive pulmonary disease, cardiovascular diseases and lung cancer. BIG3 includes 2,689 participants, where matched subcohorts were selected for comparison with the BIO-MUSE resilience cohort.

### Measures

Psychological Resilience was measured using the CD-RISC (13), containing 25 items on a 5-point Likert integer scale. Participants rate their agreement with each statement on a scale from 0 (“Not true at all”) to 4 (“True nearly all the time”). A summary score ranging from 0 to 100 is calculated, with higher scores indicating higher resilience. Internal consistency of the scale was high at baseline (Cronbach α=0.88) and follow-up (Cronbach α=0.92).

Dermatology-related QoL was assessed using the Dermatology Life Quality Index (10), containing 10 items covering different domains of QoL. Participants estimate the extent to which they agree with each statement on a 4-point Likert integer scale ranging from 0 (“Not at all”) to 3 (“Very much”). A total score is calculated on a scale of 0–30, with higher scores indicating greater impairment in QoL. Scores higher than 10 indicate the patient’s life was severely affected by their skin disease.

The severity of itch (i.e. pruritus) and sleep disturbance was measured using the 11-point Numerical Rating Scales (NRS), ranging from 0 (“no itch/sleep disturbance”) to 10 (“worst imaginable itch/sleep disturbance”). Participants rate their itch severity and sleep problems over the last 24 h.

### Data analyses

CD-RISC scores were calculated if the patient answered at least 19 of 25 items (13), and DLQI scores were calculated if the patient answered at least 9 of 10 items (10). Means and standard deviations were calculated for all measures at baseline and follow-up. Spearman correlations were conducted between the measures at both time points. Within-group differences were evaluated using the paired *t*-tests (complete cohort) and the Wilcoxon signed-rank test (subcohorts). In contrast, differences between groups were assessed using the Mann–Whitney *U* test.

CD-RISC scores in the BIO-MUSE resilience cohort were compared with the scores from the BIG3 cohort. Aside from the complete BIG3 cohort, we created sub-cohorts depending on self-reported disease status: (*i*) BIG3 participants who at some point had cancer, (*ii*) participants who never had cancer, (*iii*) BIG3 participants with skin disease (i.e. eczema and/or psoriasis) and (*iv*) BIG3 participants without any diseases (i.e. free from skin diseases, cancer or any other diagnosed conditions). To ensure sociodemographic comparability with the BIO-MUSE cohort, we utilized an iterative weighted sampling approach (18). Random subsamples from the BIG3 cohort and subcohorts were created to match the age (70 years) and gender (60% male) distribution of the BIO-MUSE resilience cohort. As the BIG3 study was designed to enroll more smokers than in the general population, and the smoking status of the BIO-MUSE resilience cohort was unknown, the subsamples were also matched to the general distribution of smokers in the Swedish population. According to population data, 7% of Swedish residents aged 65–84 smoked daily or occasionally in 2024 (19).

Subsamples of the predefined sample size were drawn without replacement in each iteration, i.e. each individual was selected only once. The characteristics of the drawn subsample were assessed against the predefined target criteria. The target criteria were allowed to deviate by 2.5% for gender, 2.5 years for age and 1.5% for smoking status. After each iteration, the weights of individuals were adjusted. The iterative process continued until a subsample was drawn that met all criteria within the prespecified range. The means and standard deviations of the CD-RISC were then calculated. These were compared to the BIO-MUSE cohort using unequal-variance *t*-tests for independent samples.

Exploratory analyses were conducted on selected DLQI and CD-RISC items. DLQI items measuring emotional and social functioning were explored, i.e. item 2 (“feeling embarrassed/ self-conscious”), item 5 (“problems in social activities”) and item 8 (“relational problems”). Spearman correlations between these items at baseline and CD-RISC at baseline and follow-up were conducted. CD-RISC items relating to emotional regulation and social relationships were selected, i.e. item 2 (“having secure relationships”), item 13 (“knowing where to turn for help”) and item 19 (“ability to handle unpleasant feelings”). These items at baseline were correlated with DLQI, itch severity and sleep disturbance at baseline and follow-up.

In all analyses, *p*-values of<0.05 were considered the threshold for significance. Correlation coefficients were deemed weak to values<0.4, moderate if 0.4–0.6, and strong if >0.6 (20). Analyses were conducted using R version 4.2.2 (21) and IBM SPSS Statistics (22).

## RESULTS

### Patient characteristics

**Fig. 1.**
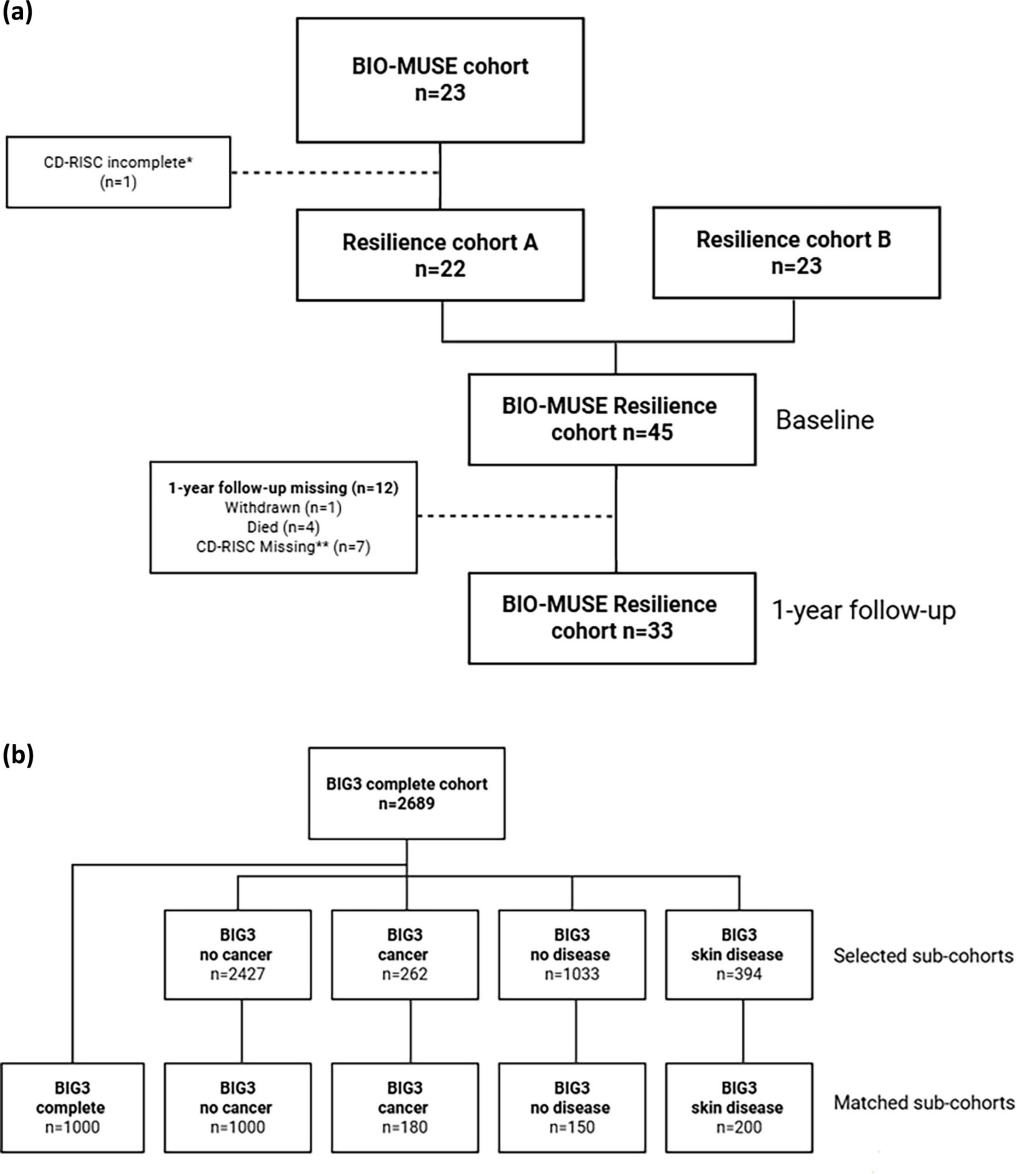
BIO-MUSE and BIG3 cohort overviews: (a) Flowchart of the patient enrolment and patient number in each resilience subcohort and in the combined BIO-MUSE resilience cohort. (b) Flowchart of the number of patients in each BIG3 subcohort. *CD-RISC incomplete refers to the fact that it is not evaluable. ** CD-RISC missing means that the 1-year follow-up has not yet been reached for the patients.

Of the 23 patients enrolled in the complete BIO-MUSE study, one did not complete the CD-RISC; thus, 22 patients could be included in the *resilience cohort A*. From the *resilience cohort B*, 23 patients were included. In total, 45 MF patients were included in the *BIO-MUSE resilience cohort*, and 33 of these patients could be assessed at the 1-year follow-up (Table I and Fig. 1a). Patient characteristics are presented in Table I. In the BIO-MUSE resilience cohort, 39 of 45 patients were presumed to have early-stage disease (IA-IIA) at baseline, and all patients (*n*=33) had early-stage disease at 1-year follow-up. The date of diagnosis was available only for resilience cohort A, and these patients had a mean disease duration of 7 years at baseline.

**Table I.** Patient characteristics of BIO-MUSE patient cohorts

Characteristics	Resilience cohort A (*n*=22*)*	Resilience cohort B (*n*=23)	BIO-MUSE Resilience cohort (*n*=45)
Age at baseline (years), mean (range)	69 (31–87)	71 (51–89)	70 (31–89)
Sex, male *n* (%)	12 (55%)	15 (65%)	27 (60%)
Disease duration at baseline (years), mean (range)	7 (0.6–16.4)	–	–
1-year follow-up (*n*)	11	22	33
Stage at diagnosis (n) IA-IIA IIB-IIIB IVA1-IVB. Missing	16 4 1 1	23	16 4 1 24
Stage at baseline (n) IA-IIA IIB-IIIB IVA1-IVB	16 5 1	23^*^ 0 0	39 5 1
Stage at 1-year follow-up (*n*) IA-IIA IIB-IIIB IVA1-IVB	11 0 0	22^*^ 0 0	33 0 0

*The exact stage of these patients was not available, and an early stage (IA–IIA) was assumed.

For the BIG3 cohort, patient characteristics, including age, gender and smoking habits, of the 5 subcohorts, created using iterative sampling with different disease status, closely match those of the BIO-MUSE resilience cohort (Table II and Fig. 1b).

**Table II.** Patient characteristics of the unmatched BIG3 complete cohort and the BIG3 subcohorts matched with BIO-MUSE resilience cohort

Characteristics	BIG3 complete cohort	BIG3 complete matched	BIG3 no cancer matched	BIG3 cancer matched	BIG3 no disease matched	BIG3 Eczema and/or psoriasis matched
Participants (*n*)	2689	1000	1000	180	150	200
Age (years), mean	63	68.5	68.4	68.4	68.5	67.5
Sex, male (%)	50	60	60	59	59	59
Smokers (%)	20	7	7.4	7.2	6.7	8.5

### Descriptive statistics

**Fig. 2.**
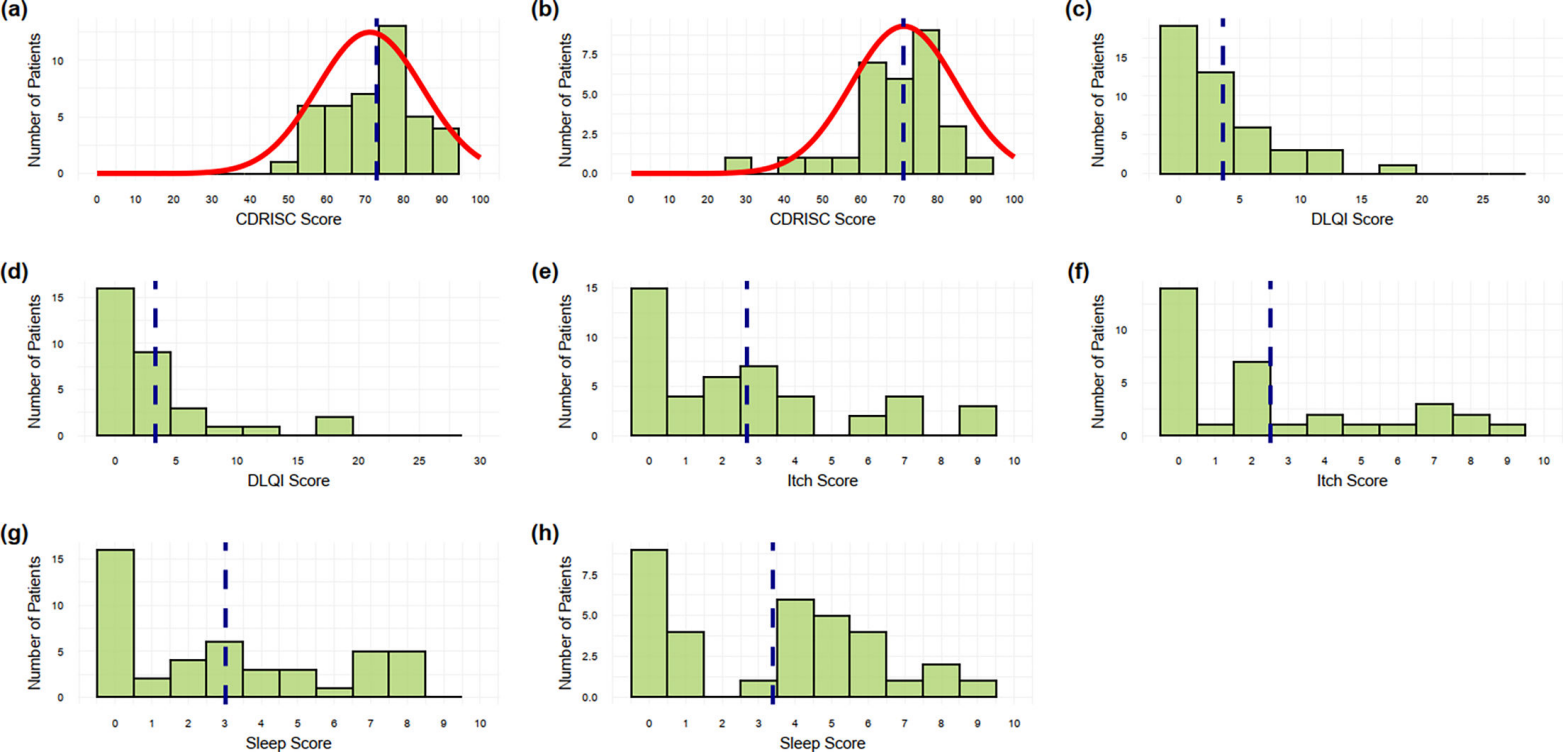
Distribution in the BIO-MUSE resilience cohort of CD-RISC score, at baseline. (a) and 1-year follow-up (b). DLQI at baseline (c) and 1-year follow-up (d). Itch severity at baseline (e) and 1-year follow-up (f). Sleep problems at baseline (g) and 1-year follow-up (h). The red line represents the normal (Gaussian) distribution curve. Blue lines represent means.

Means and standard deviations on all study measures at both baseline (BL) and 1-year follow-up (1-y FU) for the BIO-MUSE cohorts are presented in Table III. Resilience was relatively high, remained stable over time and was relatively normally distributed (Fig. 2a–b). Dermatology-related QoL was high in both subcohorts (i.e. low DLQI scores), and the distribution of DLQI was positively skewed (Fig. 2c–2d). Patients also reported low itch severity and few sleep problems at baseline and 1 year after baseline, with many reporting no itch or sleep problems (Fig. 2e–h). Patients’ scores did not change significantly over time, and there were no differences between the 2 subcohorts (all *ps*>0.05) (Table III).

**Table III.** Means and standard deviations, in the 2 resilience subcohorts and the BIO-MUSE resilience cohort, of CD-RISC, DLQI, itch severity and sleep problems at baseline and 1-year follow-up

Measures	Resilience cohort A (*n*=22) Mean (SD)	Resilience cohort B (*n*=23) Mean (SD)	BIO-MUSE Resilience cohort (*n*=45) Mean (SD)
CD-RISC BL	74.12 (10.27)	71.71 (13.07)	72.94 (11.64)
CD-RISC 1-y FU	73.22 (10.08)	70.15 (15.46)	71.20 (13.75)
DLQI BL	3.95 (4.15)	3.26 (4.01)	3.60 (4.05)
DLQI 1-y FU	3.91 (6.09)	3.00 (4.38)	3.31 (4.95)
Itch severity BL	3.36 (3.09)	2.00 (2.37)	2.67 (2.80)
Itch severity 1-y FU	3.36 (3.56)	2.09 (2.58)	2.52 (2.95)
Sleep problems BL	2.50 (2.87)	3.52 (3.01)	3.02 (2.96)
Sleep problems 1-y FU	3.27 (3.29)	3.45 (2.65)	3.39 (2.83)

No statistical differences were seen between the subcohorts or the 2 timepoints (*p-value*>0.05).

SD: standard deviation.

In a comparison of early- vs advanced-stage disease in the BIO-MUSE resilience cohort at baseline, there was no significant difference for any of the study measures (Table SI).

### Resilience in BIG3 and BIO-MUSE cohorts

Among the subcohorts from the BIG3 cohort, created using the iterative weighted sampling approach, the lowest score was found in the subcohort with eczema and/or psoriasis, and the highest in patients with no disease (i.e. healthy control group), all having a lower score than the BIO-MUSE MF patients (Table IV). BIO-MUSE patients’ resilience scores, defined at baseline, were significantly higher than those in the subcohort representing the complete BIG3 cohort, participants without cancer and participants with eczema and/or psoriasis (Table IV).

**Table IV.** Comparisons of resilience scores between the matched BIG3 sub-cohorts and the BIOMUSE resilience cohort at baseline (*n*=45, CD-RISC score=72.94)

	*n*	CD-RISC Mean	CD-RISC Std	*t*	*df*	*p-*value	CI (95%)
Complete BIG3	1,000	69.08	13.00	2.17	49	.03	0.28; 7.44
Cancer	180	69.98	12.15	1.51	70	.13	−0.94; 6.86
No cancer	1,000	68.41	12.84	2.54	49	.01	0.95; 8.11
Eczema and/or psoriasis	200	67.00	12.57	3.05	69	.003	2.05; 9.83
No disease	150	70.15	12.03	1.40	74	.17	−1.18; 6.76

CI: confidence interval; df: degrees of freedom; Std: standard deviation; t: t-test.

### Correlations between study measures

Resilience did not correlate with any other study measures (Table V). The DLQI index showed a strong positive association with itch severity, especially at one-year follow-up, and a weak positive association with the number of sleep problems, indicating that patients experienced lower QoL with greater itch severity and more sleep problems. The severity of itch and sleep problems was also moderately positively associated at baseline, with a weaker association at follow-up (Table V).

**Table V.** Spearman’s correlations between all study measures on the BIO-MUSE resilience cohort

	Baseline			
	CD-RISC	DLQI	Itch severity	Sleep problems
CD-RISC	/			
DLQI	.11	/		
Itch severity	.19	.60**	/	
Sleep problems	−.02	.33**	.50**	/
	One-year Follow-up			
	CD-RISC	DLQI	Itch severity	Sleep problems
CD-RISC	/			
DLQI	–.20	/		
Itch	–.08	.80***	/	
Sleep problems	–.00	.32**	.35*	/

*Note*: *** *p*<.001; ** *p*<.01; * *p<*.05.

### Item-level correlations

**Fig. 3.**
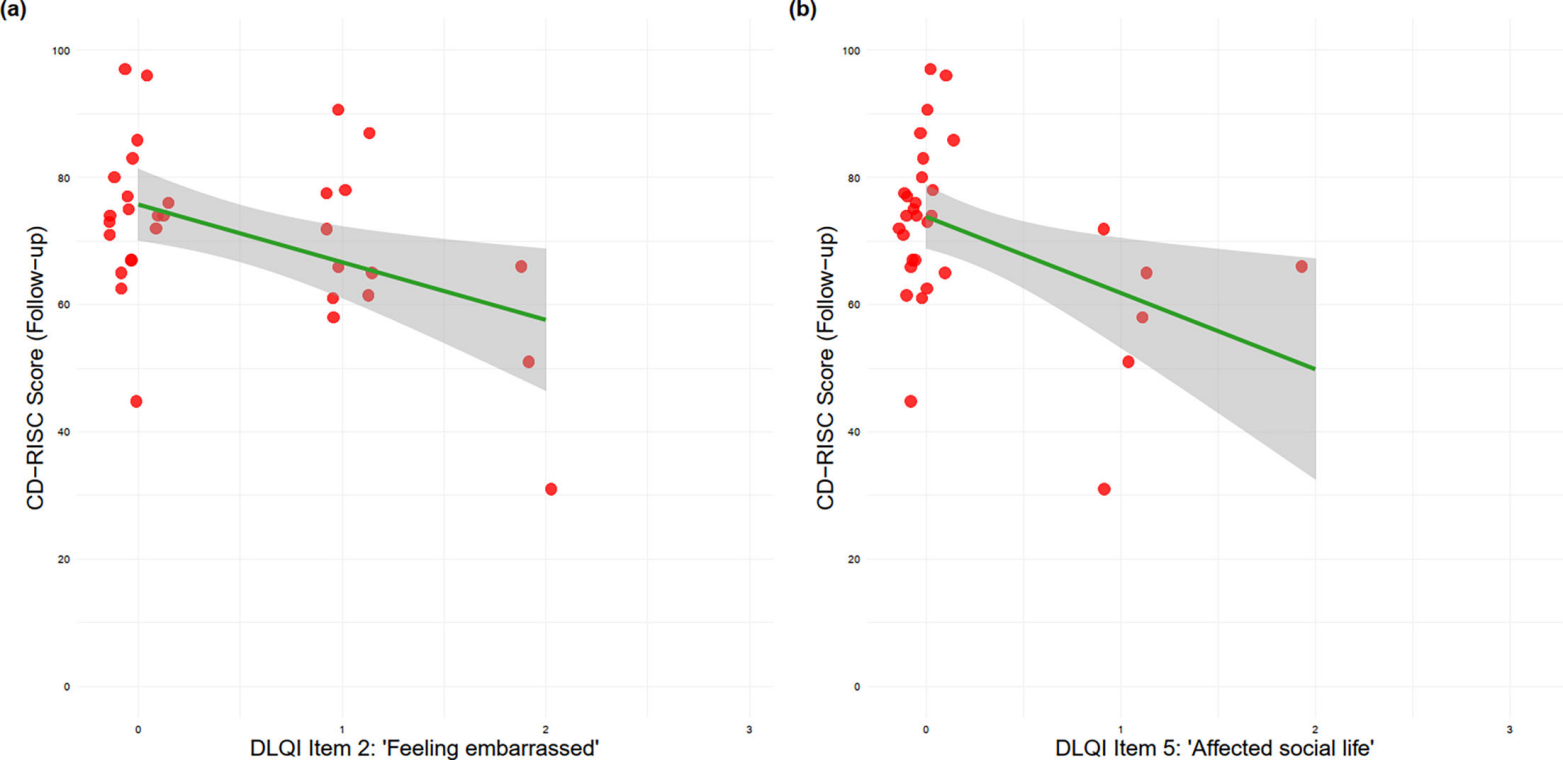
Relationship between CD-RISC at 1-year follow-up and DLQI item 2 “feeling embarrassed” at baseline (a) and DLQI item 5 “affected social life” at baseline (b).

DLQI Items 2 and 5, assessing how embarrassed or self-conscious patients feel because of their skin and how much their skin affects their social life, respectively, were negatively associated with resilience at follow-up but not at baseline (Fig. 3). DLQI item 8, assessing relational problems due to one’s skin, was not associated with resilience (Table VIa).

**Table VI.** (a) Spearman’s correlations between DLQI items 2, 5 and 8 at baseline, and CD-RISC at baseline and one-year follow-up (1-y FU). (b) Spearman’s correlations between CD-RISC items 2, 13 and 19 at baseline, and DLQI, Itch, Sleep and DLQI items 2, 5 and 8

(a)	DLQI_2 Feeling embarrassed	DLQI_5 Affected social life	DLQI_8 Relational problems	
CD-RISC BL	−.00	.01	.16	
CD-RISC 1-y FU	−.36*	−.48**	−.07	
(b)				
Measures	Timepoint	CD-RISC 2 Secure relationships	CD-RISC 13 Knowing where to turn for help	CD-RISC 19 Handling unpleasant feelings
DLQI	BL	−.35**	.13	.12
	1-y FU	−.53**	−.08	−.02
Itch	BL	−.09	.33*	.03
	1-y FU	−.46**	−.11	−.06
Sleep	BL	−.20	.30	.06
	1-y FU	−.15	.03	−.18
DLQI item 2	BL	−.36*	.12	−.05
Feeling embarrassed	1-y FU	−.42*	−.17	−.08
DLQI item 5	BL	−.11	.11	.04
Affected social life	1-y FU	−.01	−.02	.08
DLQI item 8	BL	−.18	.13	.00
Relational problems	1-y FU	−.05	−.00	−.22

Note: ** *p*<.01; * *p<*.05.

**Fig. 4.**
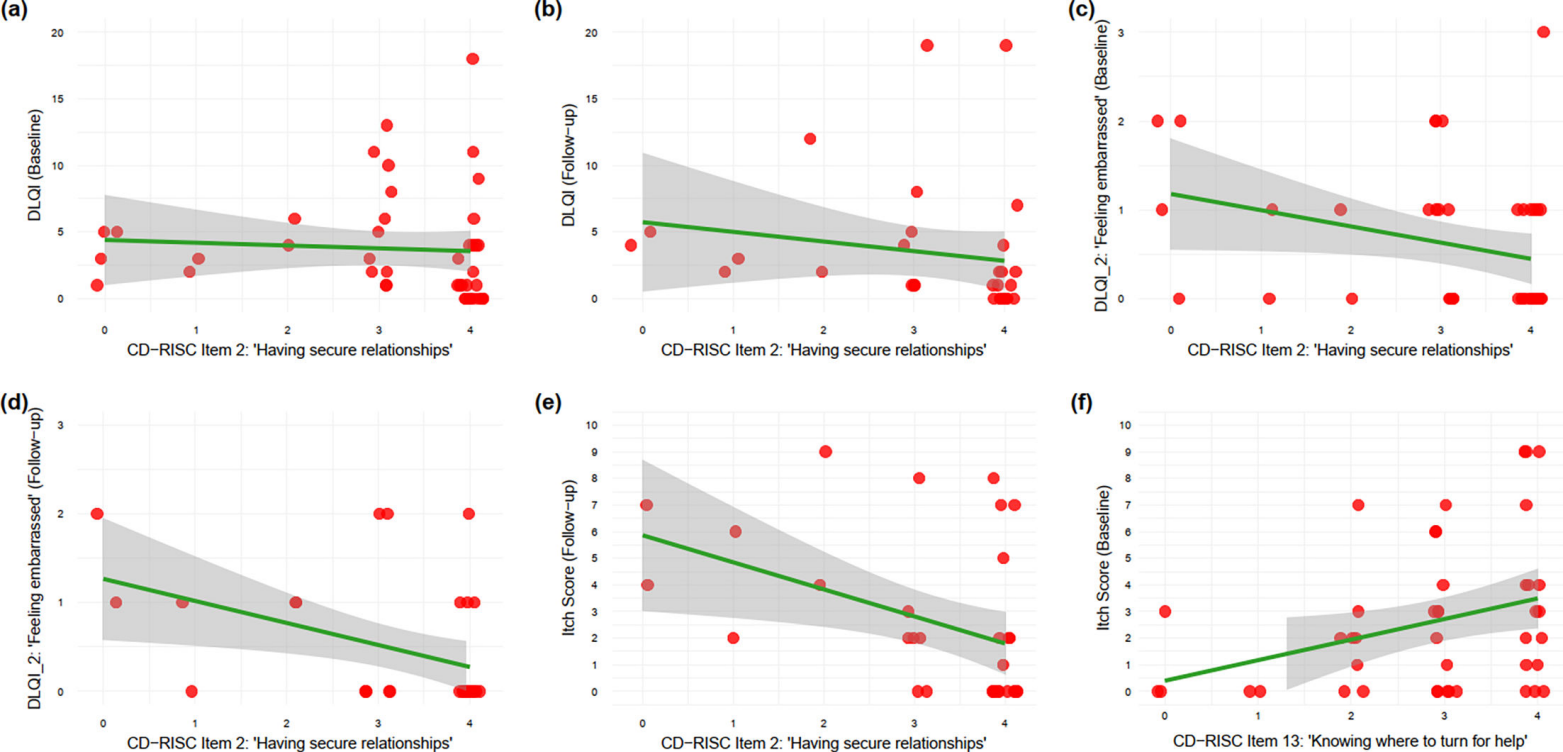
Relationship between CD-RISC item 2 “having secure relationships” at baseline and: DLQI at baseline (a); DLQI at 1-year follow-up (b); DLQI item 2 “feeling embarrassed” at baseline (c); DLQI item 2 “feeling embarrassed” at 1-year follow-up (d); Itch at 1-year follow-up (e). Relationship between CD-RISC item 13 “knowing where to turn for help” at baseline and itch at baseline (f).

CD-RISC item 2, measuring secure relationships, was negatively associated with dermatology-related QoL disturbance at both baseline and follow-up, the severity of itch at follow-up, and how embarrassed one felt about their skin. CD-RISC item 13, “Knowing where to turn for help” was positively associated with baseline itch severity (Fig. 4). Item 19, assessing ability to handle unpleasant feelings, was not associated with any measures, and sleep disturbance was not associated with any individual CD-RISC items (Table VIb).

## DISCUSSION

To our knowledge, this is the first study that investigates psychological resilience in MF patients.

In the BIO-MUSE resilience cohort, CD-RISC scores, DLQI, pruritus and sleep did not differ significantly between baseline and 1-year follow-up. Similarly, a study of breast cancer patients in southern Sweden reported a mean CD-RISC score of 70.6 at diagnosis (23, 24), with only a slight decrease observed one year later (25). Additionally, in the PROCLIPI study (26), MF patients at an early stage of disease had an improvement in QoL from diagnosis over a median follow-up time of 13 months (26). In our study, baseline assessments were conducted several years after diagnosis in most cases, suggesting that resilience in MF patients remains stable when measured further from the initial diagnosis. However, we do not know their resilience scores at diagnosis, a period often associated with high stress and uncertainty (27). It is possible that the patients in our sample had the time to adjust to their illness since the diagnosis. It is also worth noting that MF patients in the BIO-MUSE study were followed every 3 months at a highly specialized outpatient clinic, which may have contributed to stress reduction. Additionally, most patients in the study had early-stage disease.

The DLQI scores in the present study were generally low, and the mean DLQI was lower than reported in an early-stage Swedish MF patient cohort (28), but as expected, similar compared with the recently published DLQI score of 18 of the reported BIO-MUSE MF patients (29). A 2021 review concludes that dermatology-related QoL, as measured by DLQI or other scales such as SKINDEX, is lower in advanced-stage patients than in early-stage patients (9). Previously reported DLQI scores for patients with both early and advanced-stage disease ranged from 4.3 to 20 (9, 30). Comparisons between CTCL and other skin diseases, such as psoriasis and contact dermatitis, have not shown significant differences in QoL (8, 31).

CD-RISC was not significantly correlated with DLQI, itch or sleep in our patient cohort. This is surprising, as resilience has consistently been associated with HRQoL in various populations (11, 12). However, DLQI, itch severity, and sleep problems had distributions that deviated markedly from normal distribution, and low variation in scores may have contributed to these findings, as may low power due to the small patient number. Nevertheless, DLQI, itch severity and sleep disorders were positively correlated, aligning with previous studies that have suggested that itch can be used as an indicator of dermatology-specific QoL (32, 33). Comparing patients with early and advanced stages did not show a significant difference, although only 6 of the 45 patients had advanced-stage disease.

Resilience, as measured by the CD-RISC score, has been investigated in the Swedish general population cohort from the BIG3 project (15). No significant difference in CD-RISC was seen between MF patients and the matched healthy control group from the BIG3 cohort. This finding suggests that patients with MF in our cohort, mostly with early-stage disease, have a relatively high and stable resilience, comparable to that of healthy individuals. Of note, CD-RISC scores in MF patients are significantly higher than those in BIG3 participants with self-reported eczema or psoriasis. Recent publications of population cohorts in France, selecting participants with psoriasis or chronic skin diseases, revealed a lower CD-RISC score in these patients compared to a healthy control group (34, 35). These results suggest that patients with chronic skin diseases have lower CD-RISC scores than healthy controls; however, here, BIO-MUSE MF patients, despite experiencing similar chronic dermatological symptoms, have higher CD-RISC scores than those with other skin-related diseases.

Exploratory analysis revealed that dermatology-related emotional problems and social functioning problems were associated with resilience. Resilience is more closely related to psychological than to physical health-related QoL in previous studies (23), which may also be the case in MF patients. Moreover, having secure relationships, one aspect of resilience as measured by the CD-RISC, seems essential for dermatology-related QoL and itch severity, suggesting this may be a protective factor in managing the disease. The association between itch severity and knowing where to turn to for help suggests that MF patients who experienced greater itch severity gained this knowledge, enabling them to better manage their symptoms.

### Limitations

The majority of patients in the BIO-MUSE resilience cohort had early-stage disease and a low number of individuals with advanced-stage disease, suggesting that the cohort’s homogeneity masked associations between low resilience and increased disease severity. The BIO-MUSE resilience cohort is rather small, and it would be of great interest to measure resilience and QoL in a larger cohort, also including more patients with advanced-stage disease. Another limitation of this study is the short follow-up time, which may reflect the absence of differences between the timepoints. Furthermore, it would have been valuable to measure DLQI and resilience at the time of diagnosis.

### Conclusion

In this study, we have investigated psychological resilience and QoL in MF patients. No correlation was found between CD-RISC and DLQI; however, itch and sleep disorder were significantly correlated with DLQI. The BIO-MUSE MF patients exhibit a generally high QoL, compared to previously published CTCL and chronic skin disease populations. Furthermore, the patients exhibit comparable or greater resilience than both healthy controls and patients with psoriasis or eczema. DLQI and CD-RISC remain stable over one year of the disease. Altogether, the results from this study suggest that the disease- and treatment-related effects in these patients do not severely affect their QoL or psychological resilience and may indicate effective patient management in this patient cohort.

## Data Availability

The data that support the findings of this study are available from the corresponding author upon reasonable request.
