# Supplementary material for: Psychological Resilience and Quality of Life in Mycosis Fungoides Patients: A BIO-MUSE Study
Source: Acta Derm Venereol. 2026 Jun 29;106:0211. doi: 10.2340/actadv.v106.adv-2025-0211 (PMC13310848; doi:10.2340/actadv.v106.adv-2025-0211)
Supplement: Supplementary Material 1. [file ActaDv-106-0211-s0001.pdf]

Table SI. Mean and Standard Deviations of CD-RISC, DLQI, itch severity, and sleep problems at baseline in the BIO-MUSE resilience cohort patients with early-stage and advanced-stage disease. Early- and advanced-stage disease was compared using the Mann-Whitney U test and p-value <0.05 were considered a statistical significant difference.

Study measure	Early-stage (n=39)	Advanced-stage (n=6)	Mann-Whitney U test	*p-value*
	Mean (Standard deviation)			
CD-RISC	72.45 (12.01)	76.00 (9.32)	131.00	.50
DLQI	3.20 (3.54)	6.17 (6.31)	159.00	.17
Itch severity	2.41 (2.59)	4.33 (3.77)	155.50	.20
Sleep problems	2.92 (2.59)	3.67 (3.20)	130.50	.66
